# Lenvatinib complementary with radioiodine therapy for patients with advanced differentiated thyroid carcinoma: case reports and literature review

**DOI:** 10.1186/s12957-019-1626-4

**Published:** 2019-05-19

**Authors:** Nai-Wei Sheu, He-Jiun Jiang, Che-Wei Wu, Feng-Yu Chiang, Hsin-Ying Clair Chiou, Pi-Jung Hsiao

**Affiliations:** 1 Division of Endocrinology and Metabolism, Department of Internal Medicine, Kaohsiung Medical University Hospital, 100 Tzyou 1st Rd, Kaohsiung, 807 Taiwan; 2 Department of Otolaryngology-Head and Neck Surgery, Kaohsiung Medical University Hospital, Kaohsiung, Taiwan; 3 School of Medicine, College of Medicine, Kaohsiung Medical University, 100 Tzyou 1st Rd, Kaohsiung, 807 Taiwan

**Keywords:** Advanced thyroid carcinoma, Lenvatinib, Tyrosine kinase inhibitor (TKI)

## Abstract

**Background:**

The prognosis for patients with advanced differentiated thyroid carcinoma (ADTC) with disseminated distant metastases is very poor. Tyrosine kinase inhibitors targeting tumor angiogenesis have been shown to improve progression-free survival in patients with advanced thyroid carcinoma and progressive radioiodine-refractory thyroid carcinoma. Tyrosine kinase inhibitor has been reported as a successful neoadjuvant for total thyroidectomy to reduce tumor burden. However, the special indications for prompt treatment with lenvatinib as a rescue therapy to reduce tumor burden and prolong a durable response to radioiodine therapy have not been explored.

**Case presentation:**

Here, we present two ADTC cases with distant metastases who were effectively treated by total thyroidectomy combined with lenvatinib to prolong a durable response to radioiodine therapy. Case 1 was a 66-year-old male diagnosed with ADTC and disseminated brain, lung, and bone metastases. Lenvatinib was initiated via compassionate access because of rapidly progressive tumor growth even after second doses of radioiodine therapy and external beam radiation therapy for his brain metastases. The result was a durable response to lenvatinib, slowing progressive tumor growth for 3 years and allowing a third course of radioiodine therapy to treat the bone metastases. Case 2 was a 45-year-old male diagnosed with ADTC and diffuse disseminated lung metastases. Respiratory failure ensued after total thyroidectomy, requiring mandatory support by respirator. Lenvatinib was started as a rescue therapy to reduce tumor burden rapidly. The patient was successfully weaned off the respirator only 1 week after using lenvatinib. The patient was then maintained on a low dose of lenvatinib, allowing three subsequent courses of radioiodine therapy. Currently, his lung metastasis remains well controlled with decreased lung infiltrating nodules and the patient can tolerate exercise well.

**Conclusion:**

Our case experience indicated that lenvatinib has significant value as salvage therapy, reducing tumor burden, producing a durable response and maintaining quality of life. For ADTC patients with progressive life-threatening metastases, our experience suggests that lenvatinib treatment can be used as an urgent rescue therapy as well as a complement to radioiodine therapy to improve tumor eradication.

**Electronic supplementary material:**

The online version of this article (10.1186/s12957-019-1626-4) contains supplementary material, which is available to authorized users.

## Background

The incidence of thyroid cancer, particularly well-differentiated thyroid carcinoma (WDTC), shows an increasing trend globally. The overall disease-specific mortality rate is still low and has not changed in the last 40 years [1]. An incomplete response to initial therapy (persistent or recurrent structural disease) still characterizes up to 30% of patients. In the majority of cases, persistent disease remains confined to the neck, but 5–10% show distant metastasis at presentation. An additional 5–10% of patients will develop distant metastasis during follow-up. After metastasis, the prognosis is very poor for patients with tumors that are unresectable, advanced, or refractory to radioiodine therapy; median 10-year survival rates are 40–42% [2, 3].

Advanced differentiated thyroid carcinoma (ADTC) is defined by gross extrathyroidal invasion, distant metastases, radioiodine resistance, and positive 18-fluorodeoxyglucose uptake on positron emission tomography (18 FDG-PET) scan. Poorly differentiated thyroid cancer (PDTC) is characterized by infiltrative growth, necrosis, high mitosis, and vascular invasion on histopathology. Diagnosis of ADTC relies on its clinically aggressive course rather than the specific histopathology that defines PDTC [2]. About 10–20% of patients with WDTC will progress to ADTC and become resistant to standard therapy, including surgery, radioiodine therapy, TSH suppression, and chemotherapy. The prognosis for radioiodine-refractory thyroid cancer with distant metastases is very poor, with an estimated median survival of 2.5 to 3.5 years. Patients with anaplastic thyroid carcinoma (ATC) usually die within 6 months of diagnosis [1, 4]. Tyrosine kinase inhibitors (TKIs), which inhibit vascular endothelial growth factor (VEGF)-receptor signaling and tumor angiogenesis, have been used to treat advanced, progressive, and radioiodine-refractory thyroid cancers for the past 10 years. Sorafenib and lenvatinib have been approved by the US Food and Drug Administration and European Medicines Agency to improve progression-free survival in patients with ADTC or PDTC [5–7].

As a salvage therapy, TKIs should be reserved for radio-refractory patients with rapid tumor progression and severe symptoms that threaten life, according to current treatment guidelines [1, 8]. Here, we report two cases of ADTC well rescued by using lenvatinib as a neoadjuvant for further radioiodine therapy. These case reports were consented to by the patients and also approved by the Institutional Review Board of Kaohsiung Medical University Hospital.

## Case presentation

### Case 1

A 66-year old male initially presented with a huge left neck mass, shortness of breath, and gradual weight loss of 6 kg over 3 months. (Additional file 2: Table S1). His pre-operative evaluation demonstrated tracheal stricture with marked deviation by imaging studies, serum thyroglobulin (sTg) 10,470.75 ng/ml (normal < 50), and negative thyroglobulin antibody (Tg-Ab) in November 2014. Radical total thyroidectomy was done in January 2015 and verified multifocal papillary carcinoma (mixed follicular variant and focal insular/solid variant) 8.4 × 4.3 × 4.0 cm in size with lymphovascular invasion and extrathyroid extension to the muscle (Additional file 1: Figure S1), negative for BRAF V600E gene mutation. Radioiodine 200 mCi was administered in March 2015 to document stage 4c (T3N1bM1) with bilateral lung metastases. He was kept on TSH suppression and closely followed every 2~3 months. However, brain metastasis developed with a presentation of hand tremor and headache and was documented by magnetic resonance imaging (MRI). Focal neck lymph node metastases were also detected by ultrasound and echo-guided single lymph node aspiration for Tg 1522.2 ng/mL in April 2016. A second dose of radioiodine 200 mCi was administered in June 2016 and displayed a massive radioiodine-avid lesion over the bilateral lower neck, mediastinum, right occipital region, and bone (T1 and ninth rib). External beam irradiation therapy (3750 cGy divided in15 fractions) was completed focusing on the metastatic brain lesion in May 2016. However, his disease still progressed with gradual elevation of the sTg (1833.4 to 2799.3 ng/mL). Therefore, lenvatinib 24 mg was started by compassionate access in October 2016 (22nd month). The serum thyroglobulin significantly declined to 161.99 ng/ml 2 months later. Because of intolerable side effects, he was kept on a lenvatinib dosage of 5~14 mg/d. The third course of 200 mCi radioiodine therapy was then conducted in March 2018 because of a gradual increase in tumor burden detected by sTg and bone metastases. The radioiodine whole-body scan revealed interval regression of the radioiodine-avid nodal metastasis over the neck/mediastinum and right occipital region but interval progression of distant metastases over the T spines, manubrium, ischium, and iliac bone. He eventually progressed into an unavoidable status of TKIs “escape phenomenon” but is still alive.

**Fig. 1:**
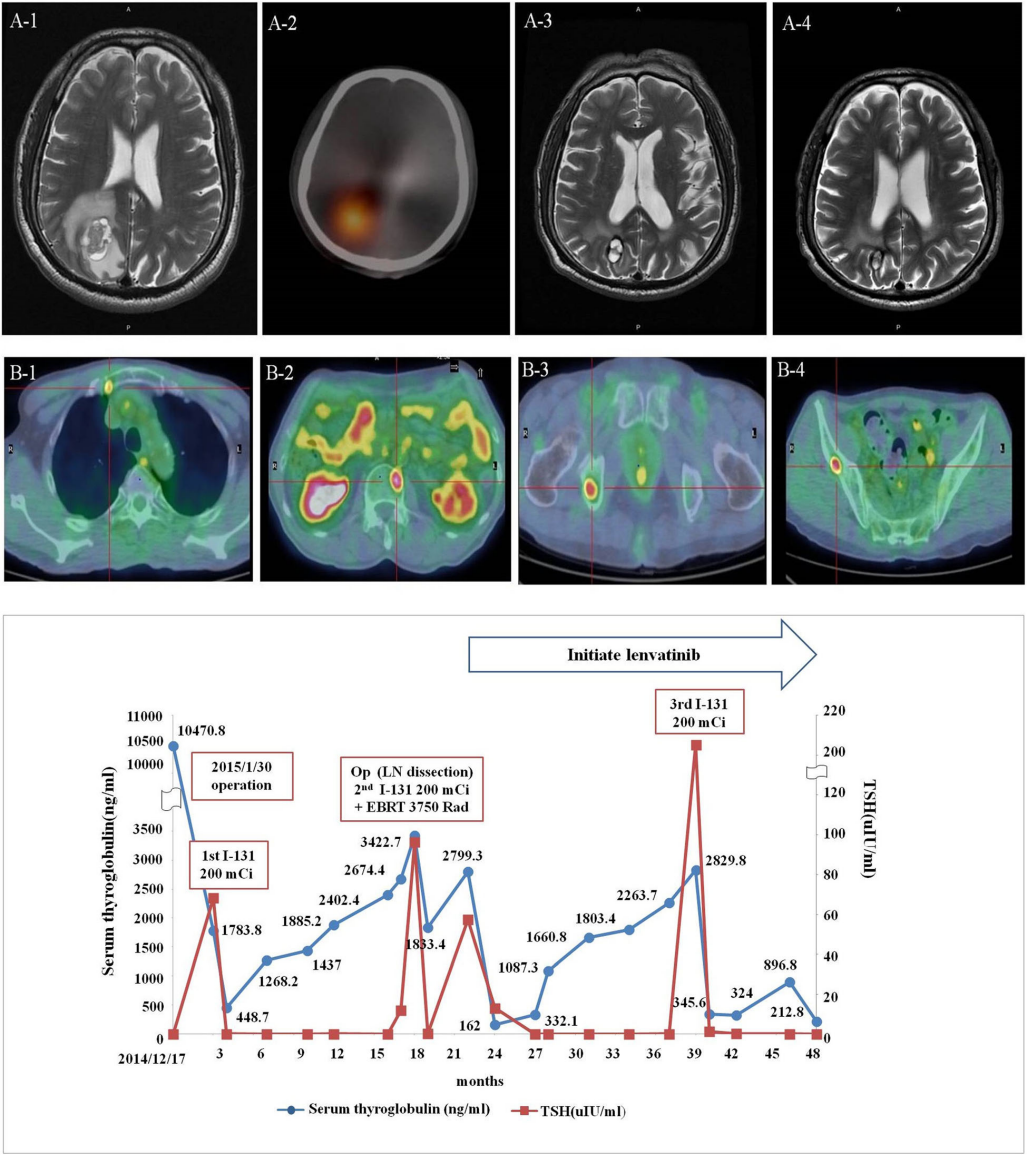
Upper panel: dynamic changes in brain and bone metastases. Hemorrhagic brain metastasis over the right occipital area was detected by brain MRI (16th month) (A-1); strong radioiodine uptake over the metastatic brain lesion was demonstrated by radioiodine whole body scan (18th month) (A-2); significant shrinkage (2.5 to 1.3 cm) of the brain metastasis after radioiodine, external beam radiation, and lenvatinib therapy, shown by brain MRI (29th month) (A-3); persistent regression of the metastatic brain lesion (39th month) (A-4). Disease progression with multiple bone metastasis was detected by 18 FDG-PET PET scan (43th month) to the right manubrium of the sternum (B-1), thoracic T4–5 spine (B-2), right ischium (B-3), and right iliac (B-4). Lower panel: the dynamic changes of serum TSH and thyroglobulin in response to various treatment modalities. The number indicates the TSH-dependent serum thyroglobulin level (ng/mL)

His treatment strategy was summarized and compared for sTg and by imaging studies over 4 years (Fig. 1). The dynamic sTg was not consistently dependent on TSH level but correlated well to the treatment modalities, including radioiodine and lenvatinib. The brain MRI clearly revealed marked shrinkage of the metastatic tumor from 2.5 to 1.3 cm after treatment. The 18FDG-PET/CT scan done in July 2018 clearly demonstrated stable disease for the brain metastasis but progression to multiple bone metastases with FDG uptake over the sternal manubrium, ribs, right ischium, iliac, left femoral shaft, and spines (L2, T4–5). A spinal MRI performed in December 2018 documented multiple metastases at the C7, T1–T6 vertebral bodies, which indicated disease progression dissociated with declining sTg.

### Case 2

**Fig. 2:**
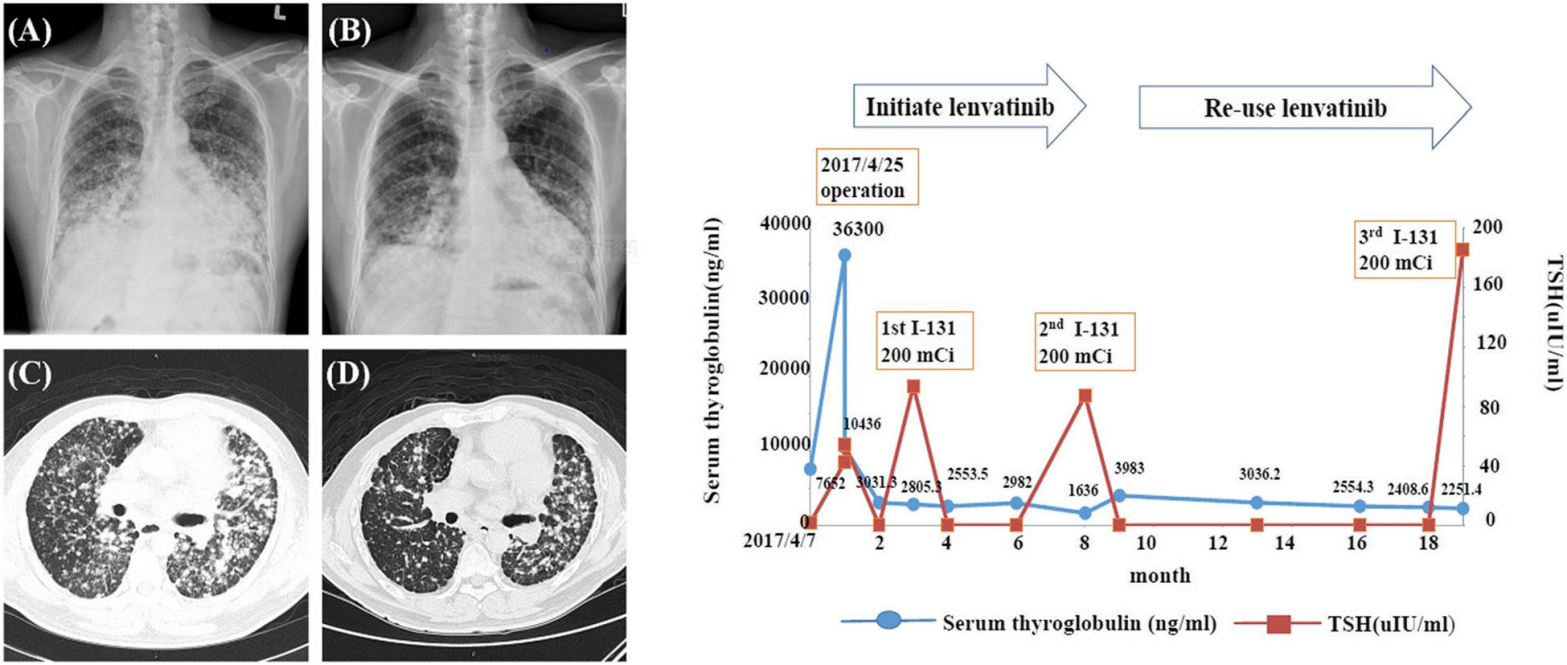
Left panel: advanced metastasis presenting with deviated trachea and diffuse disseminated nodular infiltration over both lungs in chest X-ray and CT scan (**a** and **c**) and (initial image) **a** significantly reduced intensity and range of pulmonary infiltration after treatment by total thyroidectomy, radioiodine, and lenvatinib therapy chest X-ray and CT scan (**b** and **d**) (16th month) **b** Right panel: the dynamic changes in serum TSH and thyroglobulin in response to various treatment modalities. The number indicates the TSH-independent serum thyroglobulin level (ng/mL)

A 45-year old male came for help because of a rapidly-growing neck mass combined with dyspnea, cyanosis, and gradual weight loss of 20 kg (96 to 76) within 1 year (Additional file 2: Table S2). His pre-operation evaluation revealed euthyroid status, sTg 7560 ng/mL, and negative Tg-Ab. His chest X-ray and CT scan displayed huge thyroid nodular goiters mixed with bilateral grouping lymphadenopathy and diffuse infiltrating nodules with calcification over both lungs. Wide excision of the bilateral thyroid mass with bilateral lymph node dissection was done on April 25, 2017, and documented papillary carcinoma (solid variant, sized 5.3 cm with lymphovascular invasion, extensive extrathyroid extension to adjacent organs and tissues and perineural invasion, T4aN1bM1, stage 4c) with tumor invasion to bilateral recurrent laryngeal nerve but negative for BRAF V600E gene mutation (Additional file 1: Figure S2). After operation, respiratory failure ensued and the patient was supported by respirator. Tracheostomy was done to preserve a patent airway 3 days later. Since sTg soared up to 36,300 ng/mL and the patient could not be weaned off the respirator, lenvatinib 20 mg/d was initiated on May 11, 2017. Dramatically, he was successfully weaned off the respirator 1 week later in parallel with an obvious drop of sTg to 10,436 ng/mL. He was well-trained for independent care of his tracheostomy T-tube, and radioiodine 200 mCi was scheduled 3 months later. The radioiodine-avid lesion was localized over the neck and bilateral lungs. The tracheostomy T-tube was then removed 1 month later since his condition had greatly improved, with a stationary sTg level of 2553~2982 ng/ml based on levothyroxin suppression and a low maintenance dose of 5~10 mg/d lenvatinib. Because of the persistent tumor burden over the lungs and a budget limit for the lenvatinib, a second dose of radioiodine 200 mCi was administered on January 16, 2018. However, his sTg rose again from 1636 to 3983 ng/ml even after radioiodine therapy. Lenvatinib was therefore readministered at 5 mg/d, continued to the present. Chest X-ray and chest CT both revealed a decreased intensity of the disseminated pulmonary nodular infiltration (Fig. 2). Within the treatment period (18 months), the patient has regained13 kg, can perform his daily life activities well, and tolerates a brisk walk for 40 min twice a day without shortness of breath. A third RAI 200 mCi was instituted again in December 2018 with a smooth course. His sTg level was also independent of TSH stimulation when he was maintained with lenvatinib.

## Literature review, discussion, and conclusion

The activated mitogen-activated protein kinase (MAPK) pathway, also known as the Ras-Raf-MEK-ERK pathway, is the major oncogenic driver for developing thyroid cancer, leading to altered gene expression which promotes cell proliferation, cell growth, angiogenesis, and loss of differentiation. The signaling phosphatidylinositol-3 kinase (PI3K)-Akt-mTOR pathways are also believed to promote thyroid tumor progression in ADTC or PDTC. Currently, progressive multiple hit theory is used to model the difference between WDTC, ADTC, PDTC, and ATC. WDTC is initiated by a mutation or rearrangement in the MAPK or Pax8-PPAR signaling pathways and additional mutations (PI3K/PTEN, Akt, P^53^), gene amplifications (EGFR, VEGFR), or epigenetic silencing results in progression to more aggressive ADTC, PDTC, or ATC disease [1, 2, 9]. Tyrosine kinases are responsible for regulating mitogenic signals via the phosphorylation or dephosphorylation of the proteins within the signal transduction cascade. Therapeutically, molecular targeting to inhibit tyrosine kinase can directly modulate the intracellular signal cascade and block tumor growth [10].

When PTC progresses to ADTC or PDTC, 25–50% of patients become refractory or resistant to RAI therapy. The long-term overall survival drops to 10% if distant metastases are present, particularly worse for bone or brain metastases. Treatment modalities that are suggested to control locoregional recurrence or metastatic lesions include radiofrequency thermal ablation, ethanol ablation, cryotherapy, and chemoembolization. However, it is quite challenging to manage unresectable, radioiodine-refractory or diffusely infiltrating metastatic lesions. At that point, a systemic therapy, such as TKIs, is strongly indicated [11]. TKIs have been shown to significantly decrease tumor growth, prolong tumor doubling time, and improve progression-free survival. However, no advantage in overall survival (OS) or improved life quality has been documented for TKIs because TKIs exhibit only a cytostatic rather than cytotoxic effect [11–13].

Current treatment guidelines still have no consistent consensus for who is indicated and when to initiate or stop TKI therapy. Some experts recommend TKIs for imminently threatening disease progression expected to require intervention and/or to produce morbidity or mortality in less than 6 months (such as local lesions likely to invade or obstruct airways or invade the spinal cord, or brain metastases), symptomatic disease-causing dyspnea or painful unresectable adenopathy, diffuse disease progression (such as multiple lung metastases), or multiple metastatic lesions larger than 1.5–2 cm with progressive disease over the previous 12–14 months as assessed by imaging studies [8]. Some researchers also suggest starting TKIs promptly if there is a 20% increase in the sum of the longest diameters of the target lesion over a 6-month period defined by Response Evaluation Criteria in Solid Tumors (RECIST) [14]. The National Comprehensive Cancer Network guidelines prefer the “pace of disease progression” as a treatment indicator instead of defining a tumor size or rate of change. This can be applied in clinical practice by calculating the tumor doubling time for metastatic foci measured as 1 cm in maximal diameter at baseline. A 20% increase in tumor diameter over a 6-month period approximately correlates with a diameter doubling time of 2 years, and a 40% increase in diameter means tumor growth doubling over 1 year. Based on this concept, distant metastatic lesions sized greater than 1 cm with a diameter doubling time less than 2 years could meet the criteria to initiate TKIs therapy [10, 14]. Other experts also emphasize prompt administration of TKIs without consideration of the tumor doubling time if the tumor burden is associated with dyspnea on exertion and tumor-related cachexia, or in patients with a very high overall tumor volume even if smaller-sized lesions are detected by imaging [1]. Post-operation sTg is traditionally regarded as a tumor marker to evaluate disease persistence, recurrence, or progression of WDTC. Therefore, sTg doubling time could also be a reliable surrogate marker to monitor disease activity but also requires matching with imaging assessments at appropriate time intervals [9, 11, 15].

TKI is a systemic cytostatic therapy that decelerates tumor progression in radioiodine-refractory DTC, but clinical evidence showing overall survival benefits is still lacking. A randomized, multicenter, placebo-controlled trial has demonstrated a duration of response (DOR) to lenvatinib of about 30.0 months (95% CI, 18.4–36.7) but the DOR was shorter in patients with greater tumor burden, down to 9.3 months (95% CI, 0.9–13.8) in a radioiodine-refractory DTC patient with brain metastases. The median progression-free survival (PFS) reached up to 33.1 months for lenvatinib responders vs 7.9 months for non-responders [16]. Recently, sorafenib was reported as a neoadjuvant therapy to reduce tumor volume sufficiently to allow further thyroidectomy and radioiodine therapy [17]. Thus, TKIs have changed renal cell carcinoma from unresectable to resectable in up to 20% of cases [18]. The initiation of lenvatinib treatment in our two cases met the indications of imminent life-threatening disease with brain or diffuse lung metastases and rapidly progressive tumor burden. The effective reduction of tumor burden and the slowing of tumor growth by lenvatinib exhibited a distinct complementary benefit to the cytotoxic effects of RAI therapy. Lenvatinib, targeting both the VEGF and FGF receptors, has shown better anti-angiogenic activity than sorafenib [19]. Some reports suggest lenvatinib should be the preferred first-line TKI to treat ADTC or PDTC if it is urgent to shrink metastatic lesions threatening compression of the spinal cord, trachea, or esophagus or in rapidly growing disease [11, 16, 19]. Considering the limited effect of the β emission ray in radioiodine therapy, we suggest that TKIs should not be reserved only for progressive radioiodine-refractory DTC. TKIs can be a critical rescue therapy to create an opportunity for effective tumor resection or for repeat cytotoxic radioiodine therapy to eradicate more tumor tissue. As many experts have stated, the proper application of TKI treatment to prolong life and improve patient quality of life is currently the state of the art in clinical reality [4, 20].

The tumoral escape mechanisms to TKI treatment are associated with tumor regrowth even after periods of good response to TKIs. The tumor escape phenomenon is caused by the activation of parallel proliferative signaling pathways other than the cascades inhibited by TKIs [6, 8, 11]. TKIs treatment is unable to kill tumor cells, and TKIs are usually discontinued when significant structural disease progression is documented in clinical practice [8]. However, once TKI treatment is stopped, disease progression may become even more aggressively rapid. It remains unclear how to judge the clinical value of TKIs, especially when they could significantly prolong tumor doubling time and decelerate disease progression even as structural disease progression eventually proceeds, such as in our case 1 [1, 9–11]. Debate still continues about the best time for TKI termination. From our ADTC case experiences, we prefer to use TKIs as a neoadjuvant to decelerate tumor growth and improve tumor eradication by radioiodine therapy, especially for radioiodine avid lesions. This may potentially contribute to prolonging progression-free survival and also improve overall survival.

In conclusion, we report two ADTC cases successfully rescued by lenvatinib treatment complementary with radioiodine therapy to improve quality of life. Even with widespread metastases to the brain, bone, and diffuse lung infiltration, generally considered to be hopeless in ADTC patients, our experience demonstrated a significant salvage effect by lenvatinib to reduce tumor burden, prolong a durable response to therapy, and allow further cytotoxic radioiodine therapy.

## Additional files

Additional file 1: **Figure S1.** Papillary carcinoma (mixed with follicular and focal insular/solid variant) (A) HE stain (100X), (B) HE stain (400X), (C) CK 19 stain (200X), (D) HBME-1(200X). **Figure S2.** Papillary carcinoma (solid variant); (A) HE stain (100X), (B) HE stain (400X), (C) Galectin-3 (200X), (D) HBME-1(200X). (PDF 1001 kb)

Additional file 2: **Table S1.** Treatment or event related time schedule of case 1. **Table S2.** Treatment or event related time schedule of case 2. (PDF 154 kb)

## Abbreviations

18 FDG-PET: 18-fluorodeoxyglucose uptake on positron emission tomography scan
ADTC: Advanced differentiated thyroid carcinoma
ATC: Anaplastic thyroid carcinoma
DOR: Duration of response
MAPK: Activated mitogen-activated protein kinase
MRI: Magnetic resonance imaging
OS: Overall survival
PDTC: Poorly differentiated thyroid cancer
PFS: Progression-free survival
PI3K: Phosphatidylinositol-3 kinase
RAI: Radioiodine
RECIST: Response Evaluation Criteria in Solid Tumors
sTg: Serum thyroglobulin
Tg-Ab: Thyroglobulin-antibody
TKIs: Tyrosine kinase inhibitors
VEGF: Vascular endothelial growth factor
WDTC: Well-differentiated thyroid carcinoma
