# Supplementary material for: Lenvatinib complementary with radioiodine therapy for patients with advanced differentiated thyroid carcinoma: case reports and literature review
Source: World J Surg Oncol. 2019 May 19;17:84. doi: 10.1186/s12957-019-1626-4 (PMC6525978; doi:10.1186/s12957-019-1626-4)
Supplement: Supplementary file 1 — Figure S1. Papillary carcinoma (mixed with follicular and focal insular/solid variant) (A) HE stain (100X), (B) HE stain (400X), (C) CK 19 stain (200X), (D) HBME-1(200X). Figure S2. Papillary carcinoma (solid variant); (A) HE stain (100X), (B) HE stain (400X), (C) Galectin-3 (200X), (D) HBME-1(200X). (PDF 1001 kb) [file 12957_2019_1626_MOESM1_ESM.pdf]

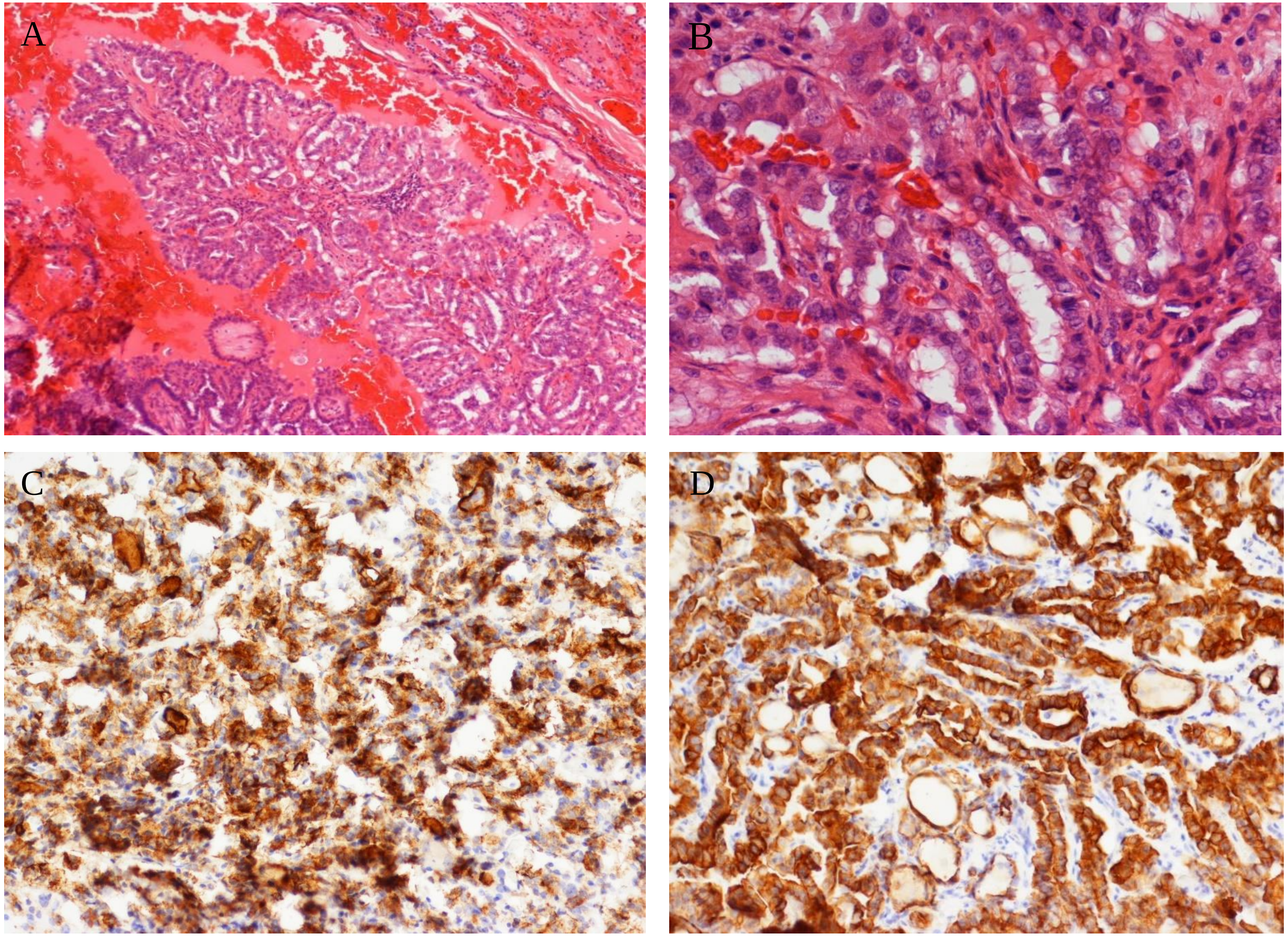

Figure S1: Papillary carcinoma (mixed with follicular and focal insular/solid variant) (A) HE stain (100X), (B) HE stain (400X), (C)CK 19 stain (200X), (D) HBME-1(200X)

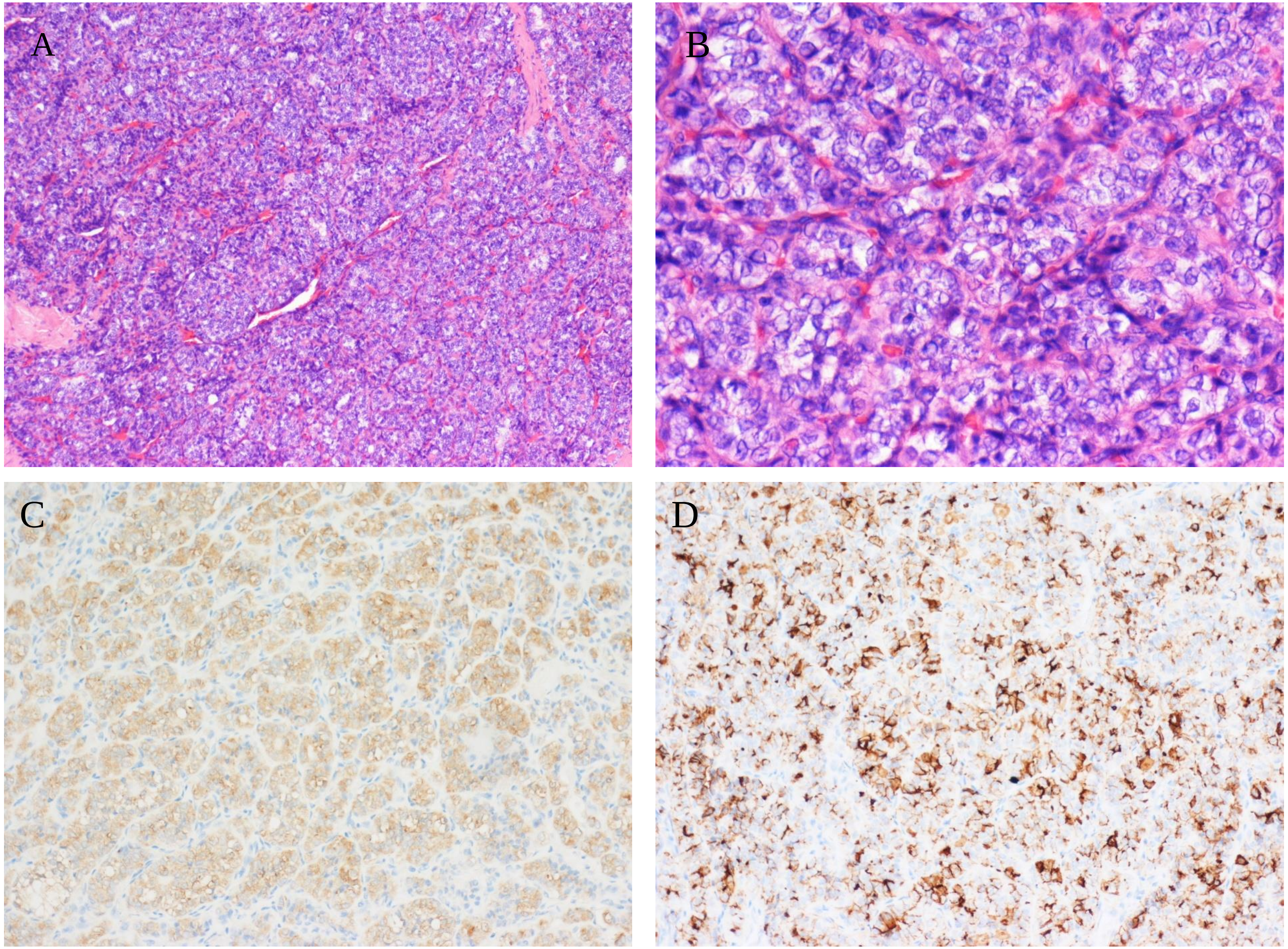

Figure S2: papillary carcinoma (solid variant); (A) HE stain (100X), (B) HE stain (400X)
(C) Galectin-3 (200X), (D) HBME-1 (200X)
