# Supplementary material for: Lenvatinib complementary with radioiodine therapy for patients with advanced differentiated thyroid carcinoma: case reports and literature review
Source: World J Surg Oncol. 2019 May 19;17:84. doi: 10.1186/s12957-019-1626-4 (PMC6525978; doi:10.1186/s12957-019-1626-4)
Supplement: Supplementary file 2 — Table S1. Treatment or event related time schedule of case 1. Table S2. Treatment or event related time schedule of case 2. (PDF 154 kb) [file 12957_2019_1626_MOESM2_ESM.pdf]

Table S1. Treatment or event related time schedule of case 1

Case 1	Time Interval (months)	TSH (uIU/ml)	Serum thyroglobulin (ng/ml)	Treatment and Event
2014/11/17	0	0.03	10470.8	Initial data, before operation
2014/12/17	0	< 0.01	4970.8	Total thyroidectomy (2015-1)
2015/3/10	3	68.17	1783.8	**1st I^{131} 200mCi** to detect lung metastases
2015/4/14	4	0.06	448.7	
2015/7/7	7	< 0.01	1268.2	
2015/10/27	10	0.03	1437.0	
2015/12/22	12	0.04	1885.2	
2016/4/19	16	< 0.01	2402.4	Lymph node dissection (2016-4), Brain metastases
2016/5/17	17	11.91	2674.4	**EBRT (3750 Rad over brain)**
2016/6/10	18	96.37	3422.7	**2nd I^{131} 200 mCi** with uptake over brain and bone metastases
2016/7/13	19	0.14	1833.4	
2016/10/11	22	57.45	2799.3	**Initiate Lenvatinib 24 mg/d**
2016/12/10	24	13.04	162.0	Intolerable side effect, downtitrate Lenvatinib dose
2017/3/2	27	< 0.01	332.1	Maintenance with Lenvatinib 10~14 mg/d
2017/4/11	28	< 0.01	1087.3	
2017/7/25	31	< 0.01	1660.8	
2017/10/7	34	< 0.01	1803.4	
2018/1/29	37	0.04	2263.7	
2018/3/9	39	145.07	2829.8	**3rd I^{131} 200 mCi**
2018/4/24	40	1.32	345.6	
2018/6/28	42	0.16	324.0	Bone scan (June), PET (July) to detect multiple bone metastases (progressive disease)
2018/10/1	46	0.04	896.8	Then uptitrate Lenvatinib dose to 20 mg/d
2018/12/7	48	0.03	212.8	

Table S2. Treatment or event related time schedule of case 2

Case 2	Time interval (months)	TSH (uIU/ml)	Serum thyroglobulin (ng/ml)	Treatment and event
2017/4/7	0	1.9	7562.0	Operation
2017/5/10	1	42.3	36300.0	Ventilator support, **Initiate Lenvatinib 20 mg/d**
2017/5/22	1	54.3	10436.0	Wean off ventilator on May 18
2017/7/1	2	0.1	3031.3	Maintenance with Lenvatinib 14 mg/d
2017/8/8	3	93.7	2803.5	**1st I^{131} 200 mCi**
2017/9/7	4	< 0.01	2553.5	Remove tracheostomy
2017/11/20	6	< 0.01	2982.0	
2018/1/16	8	87.2	1636.0	**2nd I^{131} 200 mCi,** stop Lenvatinib
2018/2/27	9	< 0.01	3983.0	Stop Lenvatinib for 1 month, then reuse Lenvatinib 5-10 mg/d
2018/6/19	13	< 0.01	3036.2	
2018/9/21	16	< 0.01	2554.3	
2018/11/15	18	< 0.01	2408.6	
2018/12/11	19	185.3	2251.4	**3rd I^{131} 200 mCi**
